# Covid-19: temporal evolution and immunization in the three epidemiological waves, Brazil, 2020–2022

**DOI:** 10.11606/s1518-8787.2022056004907

**Published:** 2022-11-18

**Authors:** Erly Catarina Moura, Juan Cortez-Escalante, Fabrício Vieira Cavalcante, Ivana Cristina de Holanda Cunha Barreto, Mauro Niskier Sanchez, Leonor Maria Pacheco Santos

**Affiliations:** I Universidade de Brasília Faculdade de Ciências da Saúde Brasília DF Brasil Universidade de Brasília. Faculdade de Ciências da Saúde. Grupo de Estudos e Pesquisa de Avaliação, Políticas, Planejamento e Gestão Participativa em Saúde. Brasília, DF, Brasil; II Organização Mundial da Saúde Organização Pan-Americana da Saúde Brasília DF Brasil Organização Mundial da Saúde. Organização Pan-Americana da Saúde. Brasília, DF, Brasil; III Universidade de Brasília Faculdade de Ciências da Saúde Brasília DF Brasil Universidade de Brasília. Faculdade de Ciências da Saúde. Programa de Pós-Graduação em Saúde Coletiva. Brasília, DF, Brasil; IV Fundação Oswaldo Cruz Ceará Eusébio CE Brasil Fundação Oswaldo Cruz Ceará. Grupo de Pesquisa em Saúde da Família e Saúde Digital. Eusébio, CE, Brasil; V Universidade de Brasília Faculdade de Ciências da Saúde Departamento de Saúde Coletiva Brasília DF Brasil Universidade de Brasília. Faculdade de Ciências da Saúde. Departamento de Saúde Coletiva. Brasília, DF, Brasil

**Keywords:** COVID-19, epidemiology, Indicators of Morbidity and Mortality, Vaccination Coverage, Health Status Disparities

## Abstract

**OBJECTIVE:**

Describe the temporal evolution of morbimortality due to Covid-19 and vaccination coverage during the health emergency in Brazil.

**METHODS:**

Number of cases and deaths due to Covid-19 were extracted from the public panel of the Brazilian Ministry of Health, according to epidemiological week (EW) and geographic region. Data on vaccines and variants were obtained, respectively, from the Information System of the National Immunization Program and the Genomic Surveillance System of SARS-CoV-2.

**RESULTS:**

Three peaks of deaths characterized the evolution of the Covid-19 pandemic: in EW 30 of 2020, in the EW 14 of 2021 and in the EW six of 2022; three case waves, starting in the North and Northeast regions, with higher rates in the third wave, mainly in the South region. Vaccination started in the epidemiological week three of 2021, rapidly reaching most of the population, particularly in the Southeast and South regions, coinciding with a reduction exclusively in the mortality rate in the third wave. Only from the beginning of the second wave, when Gama was the dominant variant, 146,718 genomes were sequenced. From the last EW of 2021, with vaccination coverage already approaching 70%, the Omicron variant caused an avalanche of cases, but with fewer deaths.

**CONCLUSIONS:**

We noticed the presence of three waves of Covid-19, as well as the effect of immunization on the reduction of mortality in the second and third waves, attributed to the Delta and Omicron variants, respectively. However, the reduction of morbidity, which peaked in the third wave during the domination of the Omicron variant, remained the same. The national and centralized command of the pandemic confrontation did not occur; thus, public administrators took the lead in their territories. The overwhelming effect of the pandemic could have been minimized, if there had been a coordinated participation of three spheres of the Brazilian Unified Health System administration, in the joint governance of the pandemic fight.

## INTRODUCTION

Since its creation and during more than three decades of existence, the Brazilian Unified Health System (SUS) has advanced, despite many challenges. More recently the Covid-19 pandemic was the main confrontation, considering the country's population size and territorial diversity.

The Brazilian Ministry of Health created the Coronavirus Panel^1^, which daily summarizes the numbers of cases and deaths due to Covid-19 throughout the country, accounted more than 30 million cases and more than 660,000 deaths until May 21, 2022, showing three propagation waves of the disease in the country.

Regarding epidemics and pandemics, public health governance should be valued, by centering national leadership mainly on the Ministry of Health, based on scientific principles and responsibility^2^. Unfortunately, the absence of those measurements in Brazil, forced other spheres of public power to assume the federal role^3^. The present study highlights the performance of the States, the Federal District and the municipalities. However, the effect of the pandemic was so devastating that, even leading this fight in their respective territories, state and municipal public administrators, either due to their extensive size or the meagre resources available, did not manage to control this outcome. Public administrators could have handled the effect of the pandemic if SUS functioned with the coordinated participation of all government spheres of the Federation^4^. The nonexistence of central coordination hindered the fight against the pandemic and postponed the implementation of Covid-19 immunization in Brazil.

This study aimed to describe the temporal evolution of cases and deaths from Covid-19 in Brazil according to the advance of vaccination coverage, by geographic regions, and the dominant variants reported in the country.

## METHODS

This is a descriptive temporal study, based on public domain secondary data. The event evaluated was Covid-19, from the first notification until the end of the state of Public Health Emergency of National Importance (ESPIN)^5^, in May 21, 2022, or the epidemiological week (EW) 20 of 2022.

Number of cases and deaths due to Covid-19 were extracted from the public panel of the Brazilian Ministry of Health, according to epidemiological week and geographic region of residence. Morbidity rates per 10,000 inhabitants and mortality per 100,000 inhabitants were calculated, considering a population estimate for the year 2020^6^.

Vaccination coverage was estimated, in percentage, by region and epidemiological week, considering individuals with full immunization (one dose or two doses, according to the type of vaccine)^7^, also using the population estimate for 2020^6^, by region and EW. The evolution of morbidity and mortality rates was plotted for the country and geographic regions as well as vaccination coverage, according to these rates.

To identify the dominant variants, the results of the sequenced genomes were extracted from the Genomic Surveillance System of SARS-CoV-2 (Severe Acute Respiratory Syndrome CoronaVirus 2) database in Brazil^8^. Data were analyzed regarding the “Main variants by sampling period” by geographic region, from February 2020 to May 2022, according to the month of samples collection, and classified as Alpha, Beta, Gamma, Delta, Omicron and others. The total number of sequenced genomes was plotted, by type of variant, according to morbidity rate and mortality rate for the country.

Since the present study is based on secondary data in the public domain, it does not require approval by an ethics committee for research with human beings^9^.

## RESULTS

The Brazilian Ministry of Health portal shows 30,945,384 cases and 666,391 deaths due to Covid-19 until May 21, 2022 (end of ESPIN), demonstrating three waves of deaths. The first wave was between February 23 (EW nine 2020) and July 25, 2020 (EW 45 2020), with 7,677 deaths weekly. The second, the longest and most lethal, occurred between November 8, 2020 (EW 46 2020) and April 10, 2021 (EW 5 2020), which ended with the triple of deaths: 21,141 in a week. The third wave was the shortest, from December 26, 2021 (EW 52 2021) to May 21, 2022, with 6,246 deaths in total (Chart).

**Chart:** Characteristics of the three epidemiological waves of Covid-19, determined by the number of deaths. Brazil, 2020–2022.

Characteristics		Epidemiological waves		
		First	Second	Third
Beginning				
	EW	9/20	46/20	52/21
	Date	July 23, 2020	Nov. 08, 2020	Dec. 26, 2021
End				
	EW	45/20	51/21	20/22
	Date	Nov. 07, 2020	Dec. 25, 2021	May 21, 2022
Duration				
	EW	37	59	23
Peak				
	EW	30/20	14/21	6/22
	Date	July 25, 2020	Apr. 10, 2021	Feb. 12, 2022
Deaths				
	Number on wave	162,269	455,379	46,046
	Number at peak	7,677	21,141	6,246
	Rate^a^ on the wave	76.5	214.7	46.0
	Rate^a^ at peak	3.6	10.0	2.9
	Rate^a^ at the end	1.1	0.3	0.1
EW (number)		4,386	7,718	2,002

EW: epidemiological week.

Source: Coronavirus Panel data^1^.

a Per 100,000 inhabitants.

**Figure 1:**
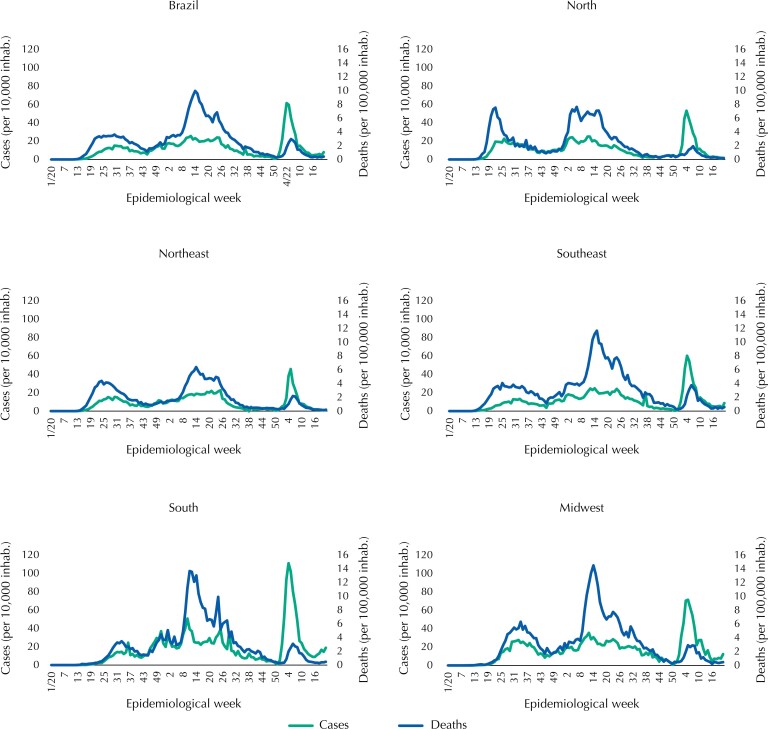
Morbidity (cases per 10,000 inhabitants) and mortality rates (deaths per 100,000 inhabitants) due to Covid-19, according to epidemiological week, country and geographic region. Brazil, 2020–2022.

Source: Coronavirus Panel data^1^.

The first wave peaked in mortality at the epidemiological week 30 of 2020, the second wave at the EW 14 of 2021, and the third in the EW six of 2022. The peaks of the waves occurred at different epidemiological weeks in the five Brazilian regions (Figure 1). The first two cases, recorded in the EW nine of 2020, occurred in the Southeast region. The evolution of the pandemic differed according to the region of the country: initially progressing in the North region, with a peak of cases during the first wave in the epidemiological week 26 of 2020, followed by the Northeast (EW 27), Midwest (EW 32), Southeast (EW 33) and, finally, the Southern (EW 36) region (Figure 1). The second wave intensified first and was more prominent in the South region, reaching more than 50 cases per 10,000 inhabitants. The third wave abruptly increased hitting all regions, with the highest rate in the South (111 cases per 10,000 inhabitants), and almost 50,000 reported cases in a single day.

Regarding mortality rates due to Covid-19 (Figure 1), the second among the three waves had the highest peak in all regions. At that time, the country had more than 15,000 deaths per week for eight weeks in a row. The North region stands out with earlier peaks in the first and second waves, followed by the Northeast and Southeast regions and, finally, the South and Midwest regions. The lowest rates occurred during the third wave, and the Southeast region had the highest mortality rates.

**Figure 2:**
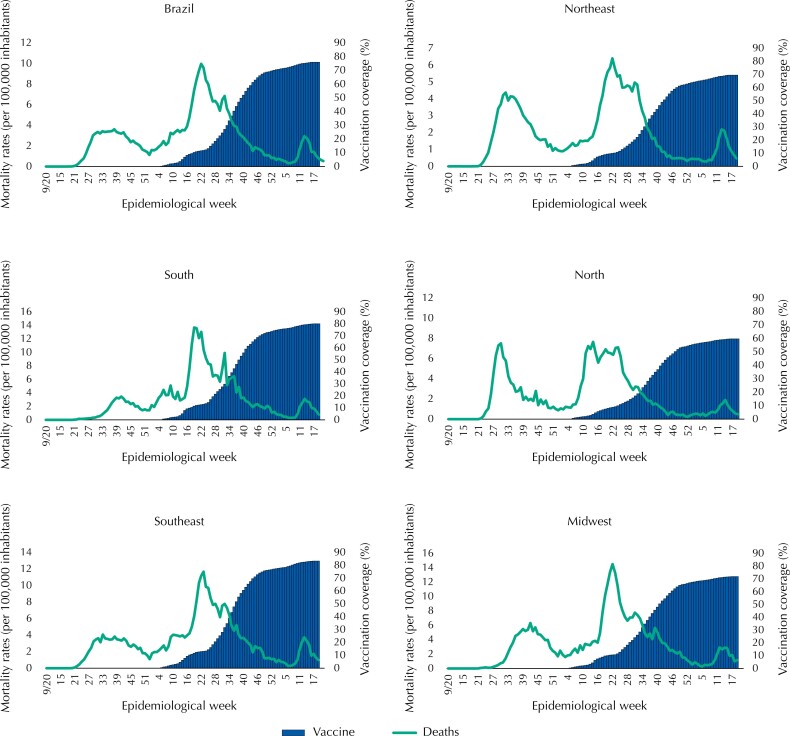
Mortality rates (deaths per 100,000 inhabitants) due to Covid-19 and vaccination coverage (%) against Covid-19, according to epidemiological week, country and geographic region. Brazil, 2020–2022.

Source: Coronavirus Panel data^1^ and the Information System of the National Immunization Program^7^.

**Figura 3:**
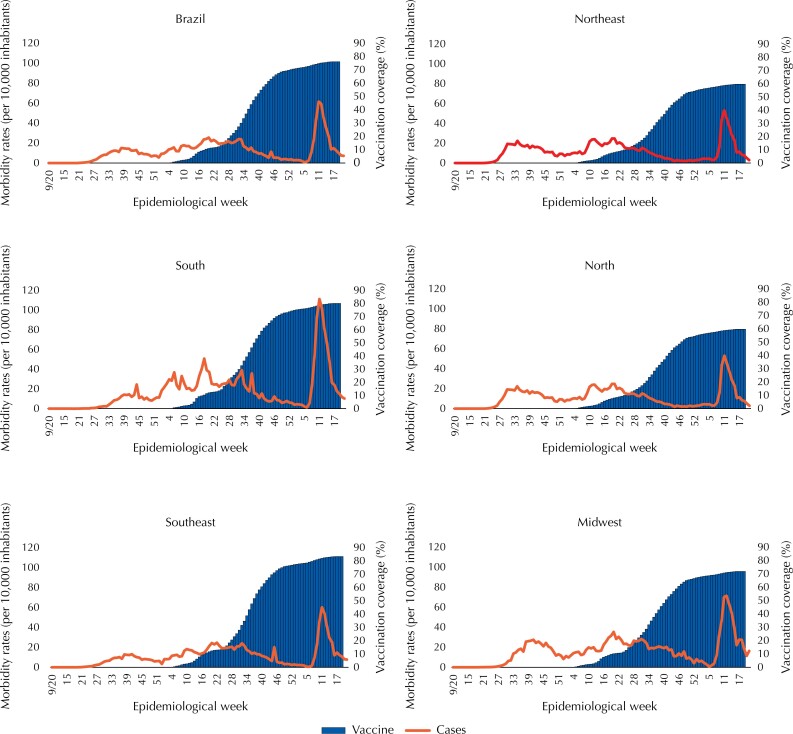
Morbidity rates (cases per 10,000 inhabitants) due to Covid-19 and vaccination coverage (%) against Covid-19, per epidemiological week, depending on the country and geographic region. Brazil, 2020–2022.

Source: Coronavirus Panel^1^ and the Information System of the National Immunization Program data^7^.

Figure 2 shows lower number of deaths with the increase in immunization against Covid-19, in the country in general and in all regions individually during the year 2021, but with a new increase in the first weeks of 2022. This scenario coincides with the increase in the number of cases (Figure 3), whose peak occurred in the epidemiological week six of 2022, regardless of vaccination coverage. Although the vaccination initiated concomitantly in all regions, its evolution was distinct, advancing faster in the Southeast and South Regions, in which the Southeast Region reached coverage of 50% around the EW 38 of 2021 and the Northern Region only in the EW 38, and by the end of this study it was almost 40% higher in the Southeast region than in the North region, achieving coverage of 83 and 60%, respectively.

**Figure 4:**
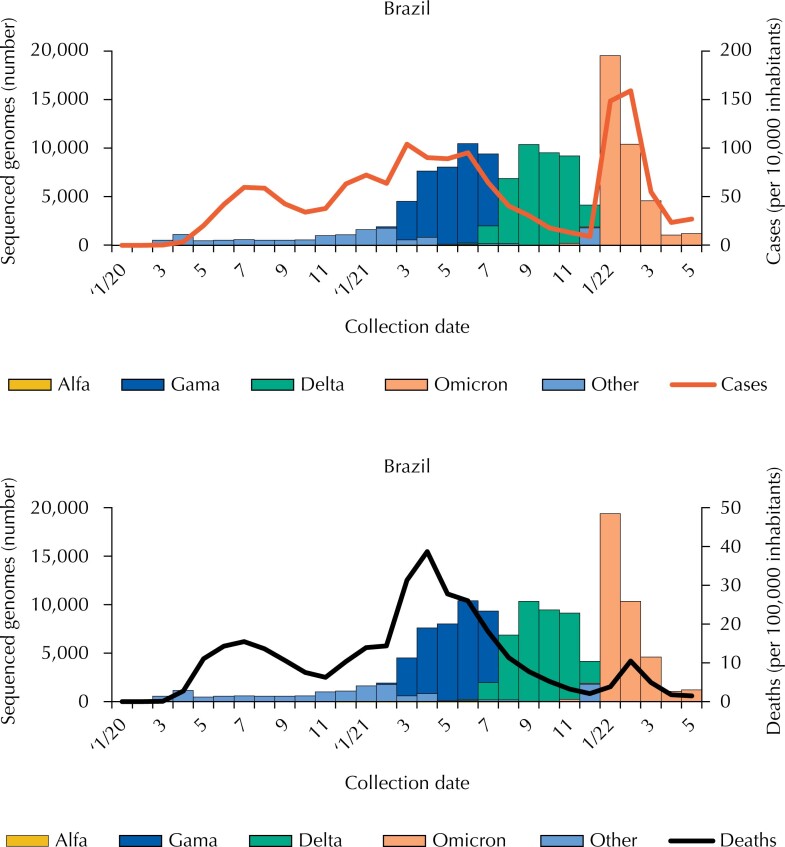
Distribution (number) of sequenced genomes, second month of collection and type of variant, morbidity rates (cases per 10,000 inhabitants) and mortality rates (per 100,000 inhabitants) due to Covid-19, Brazil, 2020–2022.

Source: Coronavirus Panel^1^ and Genomic Surveillance System of SARS-CoV-2^8^ data.

Since the start of the pandemic, 148,839 genomes have been sequenced, slowly at first, then mainly increasing from March 2021 (start of the second wave), with a higher amount in January 2022 (peak of the third wave) (Figure 4). We observed that, in 2020, practically no tests were made for the identification of strains, a period in which the Alpha variant is identified on a very small scale; in the second wave, the predominant variant was Gamma and then, Omicron. The Beta variant was not detected in Brazil. Regarding Covid-19 morbidity, the Omicron variant stands out, as for the mortality, the Gamma variant stands out.

## DISCUSSION

The data of the present study show the evolution of morbidity and mortality rates due to Covid-19 and vaccine coverage against coronavirus, unequal among the different regions of the country. Despite the identification of three waves, all of them present a spread of the pandemic in the Northern Region and, then, in the Northeast — socioeconomically disadvantaged regions. This condition impacts the vaccination coverage, which is slower in these regions, and in the Midwest, falling below the national average.

Accurate or even approximate data on the number of cases of Covid-19 in Brazil have several limitations, such as: the decision not to perform mass testing; the low availability of diagnostic tests (imported); the uncertain quality of some types of tests; the low sensitivity and specificity of the tests; the absence of registration in the information systems on the type of test performed (antigen or PCR)^10–12^, especially during the first months of the pandemic.

Since January 28, 2022, the Brazilian Health Regulatory Agency (Anvisa) authorized the registration, distribution and commercialization of self-tests for antigen detection of SARS-CoV-2^13^. Thereafter, anyone could acquire and perform their own test. However, among the tests for detecting antibodies approved in Brazil for commercialization, sensitivity range levels are low to moderate, which can generate difficulty in diagnosing infected people^10^. Self-tests are available in pharmacies, however, patients are not obligated to report positive self-tests for epidemiological surveillance, only guidance to seek medical attention is required if the result is positive^14^. In mild to moderate cases of Covid-19, many people are unlikely to seek medical care, therefore, official systems of records cannot identify them. Thus, data on the number of cases for Covid-19 became even more fragile.

The absence of genomic sequencing in the first year of the pandemic, with insufficient testing, did not cover 0.5% of the reported number of Covid-19 cases. Since no sampling process could represent the different population groups, the samples for sequencing do not reflect the national reality, acting exclusively as markers (proxy) for the identification of the dominant variants during this period.

Hospital care was in high demand, especially in the second wave when health services were overloaded, starting with the collapse in the city of Manaus^15,16^. Data from the Influenza Epidemiological Surveillance Information System (SIVEP-Gripe)^17,18^ show the highest hospital demand in the second wave, coinciding with high mortality. On the other hand, the low demand for hospitals in the third wave coincided with high vaccination coverage and the emergence of the less lethal variant — Omicron, when most cases were reported and assisted by primary and secondary care services.

The use of daily and up-to-date information on deaths enables an immediate response to the health needs imposed by the pandemic. However, the data provided by the Ministry of Health are synthesized, allowing the recording of the occurrence in a municipality different from the original, whose value is corrected in the epidemiological week *a posteriori*. Thus, the maintenance of two entries, one negative, does not give security regarding the number of occurrences of each municipality per week, also generating inaccuracy in the values per unit of the federation and region. On the other hand, the pandemic demanded speed in the information, which required immediate transparency of the data, although incomplete, but also exposes the unpreparedness regarding the quality of the information, showing mostly the need for improvement in the accuracy of the occurrence record and minimum completeness of variables such as gender, age group and municipality of residence. The pandemic also showed the possibility of implementing an agile system in a territory as large and heterogeneous as Brazil, alerting the urgency of human resources training and development of its own system for recording these data, even in the most distant locations.

Another limitation refers to the use of the 2020 population estimate, based on the 2010 census. The census in Brazil occurs every 10 years, but due to the pandemic, it did not occur in 2020. The pandemic also warns for the need of precise and safe alternatives for population counting that can be done remotely, such as several virtual visits, which surfaced during this period. However, to carry out the full census as soon as possible is urgent.

Comparing to the Mortality Information System^19^, considered the gold standard^20^, the terms of the death's registration in the Coronavirus Panel of the Brazilian Ministry of Health was delayed several weeks. Deaths due to Covid-19 started in the EW one of 2020 but were only recorded in the Panel from the EW 12, in greater number in relation to population density in the Northeast, a situation that repeated in the second wave of deaths, with higher values in the Southeast region, the most densely populated. The need to adapt health services in the first wave and high number of deaths in the second can explain these occurrences.

Moreover, the appearance of a new more lethal variant (Delta) in 2021^21^, may explain the sudden evolution of deaths in the second wave, contained by the beginning of immunization, which, although increasing, was scarce to prevent the high mortality rates in the second wave. The progressive increase in vaccination coverage in 2021 and 2022 was also unable to stop the occurrence and dispersion of cases of the Omicron variant, with high transmission power but lower lethality, reaching mostly the South region.

As other viruses, SARS-CoV-2 is also subject to mutations, by alteration in molecular structure, during the replication process. The first variant of concern (VOC) identified was Alpha (Alpha – B.1.1.7) in England; followed by Beta (Beta – B.1.351) in South Africa; Gamma (Gamma – P.1) in Brazil; Delta (Delta – B.1.617.2) in India and Omicron (Omicron – B.1.1.529) in Africa^22^. Mutations can make the virus more infectious, facilitating its entry into cells, or more transmissible, by increasing circulation, as in the case of Omicron, which quickly became the dominant variant worldwide^23,24^.

The mutation process also explains the reinfection of the disease and the drop in immunity, observed after a few months of the application of the primary vaccination schedule or the booster dose. This process contributes to the reduction of the vaccines effectiveness against infection and the decrease in sensitivity to diagnostic tests, which reinforces, from the clinical and epidemiological point of view, the need to maintain non-pharmacological measures and accelerate immunization, to reduce the circulation of the virus and the emergence of new mutations^25^. Reinfection may justify the pattern of morbidity and mortality during the third wave in this study. According to a survey conducted by the government of São Paulo, whose population has complete vaccination coverage of 88.5%, the Omicron variant caused an explosion of cases, but not deaths, in early 2022. The notification went from an average of 2,000 cases of Covid-19 daily to a peak of 14,542. However, in the same period (December 5, 2021 to February 26, 2022) the number of deaths due to Covid-19 among unvaccinated people was 26 times higher than fully immunized, showed the survey^26^. According to the Health Secretariat, in the Federal District 72% of deaths in the third wave were of unvaccinated people or those with an incomplete vaccination schedule. Complete vaccination coverage in the Federal District is 84.7%. Among the number of deaths, 85% of the people had comorbidities and the mean age was 80 years^27^.

The evolution of Covid-19 in three waves corroborates the trends observed in Europe, the Americas and Asia^28^.

The results of the present study confirm the difficulties that Brazil experienced against Covid-19. The speed with which the disease spread prevented the timely use of scientific evidence in support of government decisions^16,29^. Proposals to control the pandemic and treat the disease, without scientific support, such as the use of antibiotics, antiparasitic drugs and others, stood on the way, but then vaccines began to be tested, showing a reduction in the risk of moderate and severe complications. The beginning of vaccination was slow, with low coverage of the population at risk and amid false news about the benefit of immunization and unfounded side effects, combined with low acceptance of non-pharmacological protective measures, such as social isolation and mask use^16^.

In addition to the overload of the health system and the lack of essential inputs, such as oxygen, starting in the state of Amazonas^15,16^ and spreading rapidly throughout the rest of the country, the differentiated distribution of scarce resources made the pandemic situation even harder.

We emphasize the role of the three spheres of government in health care, concomitant with other public policies of social security and ensuring universal, full and non-discriminatory access, based on personal autonomy, the right to scientifically proven information and social control. Thus, the responsibility of the SUS, in addition to ensuring community management and epidemiology as a management tool, must ensure political-administrative decentralization with a single direction in each governmental sphere. The leadership of municipal and state public administrators in combating the Covid-19, demonstrated the construction of their own strategies to deal with the effects of the pandemic on their populations, which explains part of the regional differences.

In addition to the strengthening of local managers, leaders in their territories, the fact that articulation between the three spheres of management of the SUS could have reduced the direct and indirect effects of the pandemic on the Brazilian population stays as a lesson.
