# Supplementary material for: Covid-19: temporal evolution and immunization in the three epidemiological waves, Brazil, 2020–2022
Source: Rev Saude Publica. 2022 Nov 18;56:105. doi: 10.11606/s1518-8787.2022056004907 (PMC9749655; doi:10.11606/s1518-8787.2022056004907)
Supplement: Translation

# Translation: Covid-19: evolução temporal e imunização nas três ondas epidemiológicas, Brasil, 2020–2022

**Authors:** Erly Catarina Moura, Juan Cortez-Escalante, Fabrício Vieira Cavalcante, Ivana Cristina de Holanda Cunha Barreto, Mauro Niskier Sanchez, Leonor Maria Pacheco Santos

**Affiliations:** I Brasília DF Brasil Universidade de Brasília. Faculdade de Ciências da Saúde. Grupo de Estudos e Pesquisa de Avaliação, Políticas, Planejamento e Gestão Participativa em Saúde. Brasília, DF, Brasil; II Brasília DF Brasil Organização Mundial da Saúde. Organização Pan-Americana da Saúde. Brasília, DF, Brasil; III Brasília DF Brasil Universidade de Brasília. Faculdade de Ciências da Saúde. Programa de Pós-Graduação em Saúde Coletiva. Brasília, DF, Brasil; IV Eusébio CE Brasil Fundação Oswaldo Cruz Ceará. Grupo de Pesquisa em Saúde da Família e Saúde Digital. Eusébio, CE, Brasil; V Brasília DF Brasil Universidade de Brasília. Faculdade de Ciências da Saúde. Departamento de Saúde Coletiva. Brasília, DF, Brasil

**Keywords:** COVID-19, epidemiologia, Indicadores de Morbimortalidade, Cobertura Vacinal, Disparidades nos Níveis de Saúde

## Abstract

**OBJETIVO:**

Descrever a evolução temporal da morbimortalidade por covid-19 e da cobertura vacinal no período da emergência sanitária no Brasil.

**MÉTODOS:**

Número de casos e óbitos por covid-19 foram extraídos do painel público do Ministério da Saúde, conforme semana epidemiológica (SE) e região geográfica. Dados sobre vacinas e variantes foram obtidos, respectivamente, do Sistema de Informação do Programa Nacional de Imunizações e do Sistema de Vigilância Genômica do SARS-CoV-2.

**RESULTADOS:**

A evolução da pandemia de covid-19 caracterizou-se por três picos de óbitos: na 30ᵃ semana epidemiológica de 2020, na 14ᵃ de 2021 e na sexta de 2022; três ondas de casos, iniciando-se nas regiões Norte e Nordeste, com maiores taxas na terceira onda, principalmente na região Sul. A vacinação teve início na terceira semana epidemiológica de 2021, atingindo rapidamente a maior parte da população, particularmente nas regiões Sudeste e Sul, coincidindo com redução da taxa de mortalidade, mas não de morbidade na terceira onda. No total, 146.718 genomas foram sequenciados, mas somente a partir do início da segunda onda, na qual a variante dominante foi a Gama. A partir da última SE de 2021, quando a cobertura vacinal já se aproximava de 70%, a variante Ômicron causou uma avalanche de casos, porém com menos óbitos.

**CONCLUSÕES:**

É nítida a presença de três ondas de covid-19, bem como o efeito da imunização na redução da mortalidade na segunda e na terceira ondas, atribuídas às variantes Delta e Ômicron, respectivamente. Contudo não houve efeito na redução da morbidade, que atingiu o pico na terceira onda, na qual dominou a variante Ômicron. O comando nacional e centralizado do enfrentamento à pandemia não ocorreu; assim, os gestores locais assumiram a liderança em seus territórios. O efeito avassalador da pandemia poderia ter sido minimizado, caso houvesse a participação coordenada das três esferas de governo no SUS.

## INTRODUÇÃO

O Sistema Único de Saúde (SUS) no Brasil, desde a sua criação e durante mais de três décadas de existência, tem se caracterizado pelo avanço face a muitos desafios, destacando-se mais recentemente o enfrentamento contra a covid-19.

Nesse sentido, o Ministério da Saúde, criou o Painel Coronavírus^1^, atualizado diariamente, que sintetiza os números de casos e de óbitos por covid-19 em todo o país, contabilizando, até 21 de maio de 2022, mais de 30 milhões de casos e mais de 660 mil óbitos, evidenciando-se três ondas de propagação da doença no país.

No contexto de epidemias e/ou pandemias, a responsabilização sanitária deveria ser valorizada, com liderança nacional centralizada no Ministério da Saúde – por competência precípua – pautada em princípios científicos e responsabilidade^2^. Infelizmente, isso não ocorreu no Brasil, forçando outras esferas de poder público a assumirem o papel federal^3^. Salienta-se, aqui, o desempenho dos Estados, do Distrito Federal e dos municípios. Todavia, o efeito da pandemia foi avassalador e, mesmo tendo assumido a liderança do combate à pandemia em seus respectivos territórios, os gestores estaduais e municipais, seja pela sua extensa dimensão seja pelos parcos recursos disponíveis, não lograram controlá-la. Isso seria possível caso tivéssemos o Sistema Único de Saúde funcionando com a participação coordenada de todas as esferas de governo da Federação^4^. A inexistência de coordenação central dificultou o combate à pandemia e a implementação oportuna da imunização contra covid-19 no Brasil.

Este estudo teve como objetivo descrever a evolução temporal de casos e óbitos por covid-19 no Brasil, à luz do avanço da cobertura vacinal, conforme as regiões geográficas, e das variantes dominantes notificadas no país.

## MÉTODOS

Trata-se de estudo temporal descritivo, com dados secundários de domínio público. O evento avaliado foi a covid-19, desde a primeira notificação até o final do estado de Emergência em Saúde Pública de Importância Nacional (Espin)^5^, isto é, até 21 de maio de 2022, o que corresponde à 20ᵃ semana epidemiológica (SE) de 2022.

O número de casos e de óbitos por covid-19 foi obtido do portal do Ministério da Saúde^1^, conforme semana epidemiológica e região geográfica de residência. Taxas de morbidade por 10 mil habitantes e de mortalidade por 100 mil habitantes foram calculadas, considerando-se estimativa populacional para o ano de 2020^6^.

A cobertura vacinal foi calculada, em percentual, por região e semana epidemiológica, considerando-se indivíduos com imunização integral (uma dose ou duas doses, conforme o tipo de vacina)^7^, utilizando, também, a estimativa populacional para 2020^6^, por região e SE. A evolução das taxas de morbidade e mortalidade foi plotada para o país e regiões geográficas bem como a cobertura vacinal, conforme taxa de mortalidade e taxa de morbidade.

Para a identificação das variantes dominantes, os resultados dos genomas sequenciados foram extraídos do banco de dados da Vigilância Genônima do SARS-CoV-2 (*Severe Acute Respiratory Syndrome CoronaVirus* 2) no Brasil^8^. Foram analisados dados referentes às “Principais variantes por período de amostragem” por região, de fevereiro de 2020 a maio de 2022, conforme o mês de coleta das amostras, por região geográfica e classificadas como Alfa, Beta, Gama, Delta, Ômicron e Outra. O número total dos genomas sequenciados foi plotado, por tipo de variante, conforme taxa de morbidade e taxa de mortalidade para o país.

Por se tratar de dados secundários de domínio público, este estudo dispensa aprovação por comitê de ética em pesquisa com seres humanos^9^.

## RESULTADOS

No portal do Ministério da Saúde, foram contabilizados até 21 de maio de 2022 (final da Espin), 30.945.384 de casos de covid-19 e 666.391 óbitos, evidenciando-se claramente três ondas de óbitos. A primeira onda estendeu-se de 23 de fevereiro (9ᵃ SE 2020) a 25 de julho de 2020 (45ᵃ SE 2020), quando foram notificados 7.677 óbitos semanais. A segunda, mais longa e mais letal, ocorreu entre 8 de novembro de 2020 (46ᵃ SE 2020) e 10 de abril de 2021 (51ᵃ SE), que terminou com o triplo de óbitos: 21.141 mortes em uma semana. A terceira onda foi a mais curta, de 26 de dezembro de 2021 (52ᵃ SE 2021) a 21 de maio de 2022, na qual ocorreram 6.246 óbitos no total (Quadro).

**Quadro:** Características das três ondas epidemiológicas de covid-19, determinadas pelo número de óbitos. Brasil, 2020–2022.

Características		Ondas epidemiológicas		
		Primeira	Segunda	Terceira
Início				
	SE	9/20	46/20	52/21
	Data	23 fev 2020	08 nov 2020	26 dez 2021
Término				
	SE	45/20	51/21	20/22
	Data	07 nov 2020	25 dez 2021	21 mai 2022
Duração				
	SE	37	59	23
Pico				
	SE	30/20	14/21	6/22
	Data	25 jul 2020	10 abr 2021	12 fev 2022
Óbitos				
	Número na onda	162.269	455.379	46.046
	Número no pico	7.677	21.141	6.246
	Taxa^a^ na onda	76,5	214,7	46,0
	Taxa^a^ no pico	3,6	10,0	2,9
	Taxa^a^ ao término	1,1	0,3	0,1
	SE(número)	4.386	7.718	2.002

SE: semana epidemiológica.

Fonte: dados do Painel Coronavírus^1^.

a Por 100 mil habitantes.

A primeira onda teve pico de mortalidade na 30ᵃ semana epidemiológica de 2020; já na segunda o auge ocorreu na 14ᵃ semana de 2021 e a terceira na 6ᵃ SE de 2022. Os picos das ondas não foram coincidentes nas cinco regiões brasileiras (Figura 1). Os dois primeiros casos, registrados na 9ᵃ SE de 2020, ocorreram na região Sudeste. Todavia, a evolução da pandemia diferiu conforme a região do país, progredindo inicialmente na região Norte, com pico de casos para a primeira onda na 26ᵃ semana epidemiológica de 2020, seguida pela região Nordeste (27ᵃ SE), Centro-Oeste (32ᵃ SE), Sudeste (33ᵃ SE) e, por último, a região Sul (36ᵃ SE), conforme ilustra a Figura 1. A segunda onda recrudesceu primeiro e foi mais proeminente na região Sul, alcançando taxa maior do que 50 casos por 10 mil habitantes. A terceira onda irrompeu abruptamente atingindo todas as regiões, com maior taxa na região Sul (111 casos por 10 mil habitantes), com quase 50 mil casos notificados em um único dia.

**Figura 1:** Taxas de morbidade (casos por 10 mil habitantes) e de mortalidade (óbitos por 100 mil habitantes) por covid-19, de acordo com a semana epidemiológica, conforme o país e a região geográfica. Brasil, 2020–2022.

Fonte: dados do Painel Coronavírus^1^.

Quanto às taxas de mortalidade por covid-19 (Figura 1), também se notam três ondas, com maior pico na segunda onda em todas as regiões. Nessa época ocorreram no país mais de 15 mil óbitos por semana, durante oito semanas seguidas. Destaca-se a região Norte, com picos mais precoces na primeira e na segunda ondas, seguida pelas regiões Nordeste e Sudeste e, por último, as regiões Sul e Centro-Oeste. As menores taxas foram registradas na terceira onda, sendo que os maiores valores registrados foram registrados na região Sudeste.

Fica evidente (Figura 2) o menor número de óbitos com o aumento da imunização contra a covid-19, no país em geral e em todas as regiões individualmente durante o ano de 2021, mas com novo aumento nas primeiras semanas de 2022. Esse cenário coincide com o aumento do número de casos (Figura 3), cujo pico ocorre na 6ᵃ semana epidemiológica de 2022, independente da cobertura vacinal. Embora o lançamento da vacinação tenha sido concomitante em todas as regiões, a evolução foi distinta, mostrando avanço mais acelerado nas Regiões Sudeste e Sul, sendo que a Região Sudeste alcançou cobertura de 50% por volta da 38ᵃ semana de 2021 e a Região Norte somente na 48ᵃ, chegando ao final deste estudo com coberturas de 83 e 60%, respectivamente, isto é, quase 40% maior na região Sudeste do que na região Norte.

**Figura 2:** Taxas de mortalidade (óbitos por 100 mil habitantes) por covid-19 e cobertura vacinal (%) contra covid-19, e acordo com a semana epidemiológica, conforme o país e a região geográfica. Brasil, 2020–2022.

Fonte: dados do Painel Coronavírus^1^ e do Sistema de Informação do Programa Nacional de Imunizações^7^.

**Figura 3:** Taxas de morbidade (casos por 10 mil habitantes) por covid-19 e cobertura vacinal (%) contra covid-19, por semana epidemiológica, conforme o país e a região geográfica. Brasil, 2020–2022.

Fonte: dados do Painel Coronavírus^1^ e do Sistema de Informação do Programa Nacional de Imunizações^7^.

Desde o início da pandemia, 148.839 genomas foram sequenciados, timidamente ao início, aumentando principalmente a partir de março de 2021 (início da segunda onda), com maior quantidade em janeiro de 2022 (pico da terceira), conforme ilustra a Figura 4. Observa-se que, em 2020, praticamente não houve testes para a identificação das linhagens, período no qual é identificada, em escala muito pequena, a variante Alfa; já na segunda onda, a variante predominante foi a Gama e, na sequência, a Ômicron. A variante Beta não foi detectada no Brasil. No que diz respeito à morbidade por covid-19, destaca-se a variante Ômicron, e quanto à mortalidade a variante Gama.

**Figura 4:** Distribuição (número) dos genomas sequenciados, segundo mês de coleta e tipo de variante, taxas de morbidade (casos por 10 mil habitantes) e de mortalidade (por 100 mil habitantes) por covid-19, Brasil, 2020–2022.

Fonte: dados do Painel Coronavírus^1^ e do Sistema de Vigilância Genônima do SARS-CoV-2^8^.

## DISCUSSÃO

Os dados apresentados neste estudo mostram a evolução das taxas de morbimortalidade por covid-19, bem como da cobertura vacinal contra o coronavírus, de forma desigual, nas diferentes regiões do país. Apesar da identificação de três ondas, em todas elas se nota o alastramento da pandemia na Região Norte e, na sequência, Nordeste – regiões sabidamente mais desfavorecidas socioeconomicamente. Essa situação tem reflexos na cobertura vacinal, que também é mais lenta nessas regiões, ficando abaixo da média nacional, incluindo também a Região Centro-Oeste.

Dados precisos ou, mesmo, aproximados, sobre o número de casos de covid-19 no Brasil esbarram em várias limitações, como a decisão de não realizar testagem em massa; a baixa disponibilidade de testes diagnósticos (importados); a qualidade incerta de alguns tipos de testes; a pouca sensibilidade e especificidade dos testes; a ausência do registro, nos sistemas de informação sobre o tipo de teste realizado (antígeno ou PCR)^10–12^, em especial durante os primeiros meses da pandemia.

A partir de 28 janeiro de 2022, a Agência Nacional de Vigilância Sanitária autorizou o registro, a distribuição e a comercialização de autotestes para detecção de antígeno do SARS-CoV-2^13^. A partir de então, qualquer pessoa pôde adquirir e realizar seu próprio teste, no entanto, dentre os testes para detecção de anticorpos aprovados no Brasil para comercialização, a sensibilidade encontra-se na faixa de níveis baixos a moderados, o que pode gerar dificuldade de diagnóstico de pessoas infectadas^10^. Autotestes estão disponíveis nas farmácias, porém, não há obrigatoriedade de notificação de autotestes positivos para vigilância epidemiológica, apenas orientação para que o paciente procure assistência médica caso o resultado seja positivo^14^. Supõe-se que em casos leves a moderados de covid-19 muitas pessoas provavelmente não procurarão assistência médica, e, portanto, não poderão ser identificados em nenhum sistema oficial de registros. Desse modo, dados sobre o número de casos para covid-19 se tornaram ainda mais frágeis.

Verifica-se, ainda, a ausência de sequenciamento genômico no primeiro ano da pandemia, com testagem insuficiente, que não chegou a cobrir 0,5% do número notificado de casos por covid-19. Ademais, as amostras para sequenciamento não refletem a realidade nacional, uma vez que não houve qualquer processo de amostragem que pudesse representar os diferentes grupos populacionais, servindo exclusivamente como marcadores (*proxy*) para a identificação das variantes dominantes no decorrer desse período.

Houve alta procura por atendimento hospitalar, principalmente na segunda onda, quando os serviços de saúde ficaram sobrecarregados, iniciando com o colapso na cidade de Manaus^15,16^. Dados do Sistema de Informação da Vigilância Epidemiológica da Gripe (SIVEP-Gripe)^17,18^ mostram claramente a maior procura hospitalar na segunda onda, coincidindo com alta mortalidade. Já a baixa procura por hospitais na terceira onda, coincidiu com altas coberturas vacinais e com o surgimento da variante menos letal – Ômicron – quando a maior parte dos casos foi notificada e atendida pelos serviços de atenção primária e secundária.

A utilização de informações diárias e atualizadas sobre óbitos possibilita responder de imediato às necessidades sanitárias impostas pela pandemia. Todavia, os dados disponibilizados pelo Ministério da Saúde são sintetizados, permitindo inclusive o registro da ocorrência em município distinto do original, cujo valor é corrigido na semana epidemiológica *a posteriori*. Assim, a manutenção de duas entradas, uma delas negativa, não dá segurança quanto ao número de ocorrências de cada município por semana, o que gera imprecisão também nos valores por unidade da federação e região. Por outro lado, a pandemia demandou rapidez na informação, o que exigia transparência imediata dos dados, ainda que incompletos, mas também expõe o despreparo quanto à qualidade da informação, mostrando além da necessidade de agilização, necessidade de aperfeiçoamento na precisão do registro da ocorrência e completude mínima de variáveis como sexo, faixa etária e município de residência. Positivamente, mostrou a possibilidade de implantação de um sistema ágil num território tão grande e heterogêneo como o Brasil, alertando para a urgência de treinamento de recursos humanos e desenvolvimento de um sistema próprio para registro desses dados, mesmo nos locais mais distantes.

Outra limitação se refere ao uso da estimativa populacional de 2020, baseada no censo de 2010. O Brasil tem sido recenseado a cada 10 anos, mas em 2020 não houve censo, por conta da pandemia. A pandemia também alerta para a necessidade de alternativas precisas e seguras para a contagem populacional que possam ser realizadas à distância, a exemplo de vários atendimentos virtuais, que afloraram nesse período. De qualquer modo, urge a realização do censo completo o mais brevemente possível.

Comparativamente, ao Sistema de Informações sobre Mortalidade^19^, considerado padrão-ouro^20^, nota-se atraso de várias semanas quanto ao registro de óbitos no Painel do Ministério da Saúde. De modo geral, as mortes por covid-19 tiveram início na primeira semana epidemiológica de 2020, mas só foram registradas no Painel a partir da 12ᵃ semana, em maior número em relação à densidade populacional no Nordeste, situação que se repetiu na segunda onda de óbitos, com maiores valores na região Sudeste, a mais densamente povoada. Essas ocorrências podem ser explicadas pela necessidade de adaptação dos serviços de saúde na primeira onda e alto número de óbitos na segunda.

Ademais, o aparecimento de uma nova variante mais letal (Delta) em 2021^21^, pode explicar a brusca evolução de óbitos na segunda onda, contida pelo início da imunização, que, apesar de crescente, não foi suficiente para impedir as elevadas taxas de mortalidade na segunda onda. O aumento progressivo nas coberturas vacinais, em 2021 e 2022, também não foram capazes de deter a ocorrência e dispersão de casos da variante Ômicron, de alto poder de transmissão, mas com menor letalidade, atingindo mais a região Sul.

Como os demais vírus, o SARS-CoV-2 também está sujeito a mutações, por alteração na estrutura molecular, durante o processo de replicação. A primeira variante de preocupação (VOC – *variant of concern*) identificada foi a Alfa (Alpha – B.1.1.7) na Inglaterra; seguida pela Beta (Beta – B.1.351) na África do Sul; Gama (Gamma – P.1) no Brasil; Delta (Delta – B.1.617.2) na Índia e Ômicron (Omicron – B.1.1.529) na África^22^. As mutações podem tornar o vírus mais infeccioso, facilitando sua entrada nas células, ou mais transmissível, pelo aumento da circulação, como no caso da Ômicron, que rapidamente se tornou a variante dominante no mundo todo^23,24^.

O processo de mutação também explica a reinfeção da doença, conjuntamente com a queda de imunidade que se observa após alguns meses da aplicação do esquema vacinal primário ou da dose de reforço. Também contribui para a redução da efetividade das vacinas contra a infecção e a diminuição da sensibilidade aos testes diagnósticos, o que reforça, do ponto de vista clínico e epidemiológico, a necessidade de manutenção das medidas não farmacológicas e aceleração da imunização, para reduzir a circulação do vírus e o aparecimento de novas mutações^25^. A reinfecção pode justificar o padrão de morbimortalidade observado durante a terceira onda neste presente estudo. Segundo levantamento realizado pelo governo de São Paulo, cuja população apresenta cobertura vacinal completa de 88,5%, a variante Ômicron causou uma explosão de casos, mas não de óbitos, no início de 2022. A notificação passou de uma média de dois mil casos diários de covid-19, para um pico de 14.542 casos por dia. Contudo, no mesmo período (5 de dezembro de 2021 a 26 de fevereiro de 2022) o número de óbitos por covid-19 entre pessoas não vacinadas foi 26 vezes maior do que entre as pessoas completamente imunizadas, revelou o levantamento^26^. Situação semelhante foi registrada no Distrito Federal, onde 72% dos óbitos na terceira onda foram de pessoas não vacinadas ou com esquema incompleto, revelou a Secretaria de Saúde. A cobertura vacinal completa no DF é de 84,7%. Entre os que foram a óbito 85% tinham comorbidades e idade média de 80 anos^27^.

De modo geral, a evolução do covid-19 em três ondas corrobora as tendências observadas na Europa, nas Américas e na Ásia^28^.

Os resultados do presente estudo confirmam as dificuldades que o Brasil passou no enfrentamento à covid-19. A rapidez com que a doença se disseminou não possibilitou o uso em tempo oportuno de evidências científicas no apoio às decisões governamentais^16,29^. Propostas de controle da pandemia e tratamento da doença, sem sustentação científica, ficaram no caminho, como uso de antibióticos, antiparasitários e outros, deram lugar às vacinas, que começaram a ser testadas, mostrando redução no risco de complicações moderadas e graves. O início da vacinação não foi ágil, com baixa cobertura da população em risco e em meio a notícias falsas sobre o benefício da imunização e sobre infundados efeitos colaterais, aliado à baixa aceitação a medidas não farmacológicas de proteção, como isolamento social e uso de máscara^16^.

Além da sobrecarga do sistema de saúde e da falta de insumos essenciais, como oxigênio, iniciando no estado do Amazonas^15,16^ e se espalhando vertiginosamente pelo restante do país, a distribuição diferenciada dos parcos recursos dificultou mais ainda a situação pandêmica.

Aqui, cabe ressaltar o papel das três esferas de governo na assistência à saúde, concomitante às demais políticas públicas de seguridade social e garantindo o acesso universal, integral e não discriminatório, pautado na autonomia pessoal, no direito às informações cientificamente comprovadas e no controle social. Assim, a responsabilidade do SUS, além de garantir a gestão comunitária e a epidemiologia como instrumento de gestão, deve assegurar a descentralização política-administrativa com direção única em cada esfera governamental. É importante destacar a liderança no combate à covid-19 dos gestores municipais e estaduais, na construção de estratégias próprias para lidar com os efeitos da pandemia nas suas populações, o que explica parte das diferenças regionais.

Além do fortalecimento dos gestores locais, na liderança em seus territórios, fica como aprendizado que a articulação entre as três esferas de gestão do SUS poderia ter reduzido os efeitos diretos e indiretos da pandemia sobre a população brasileira.
